# The Use of Platelet-Rich Plasma Augmentation in Meniscus Repair Results in a Lower Failure Rate than in the Control Group: A Systematic Review From Meta-analysis

**DOI:** 10.1016/j.asmr.2024.100934

**Published:** 2024-04-09

**Authors:** Muhammad Sakti, Idrus Andi Paturusi, Leonard Christianto Singjie, Samuel Andi Kusuma

**Affiliations:** a Department of Orthopedic and Traumatology, Faculty of Medicine, Hasanuddin University, Makassar, Indonesia; b Atma Jaya Hospital, Jakarta, Indonesia

## Abstract

**Purpose:**

To investigate the efficacy of platelet-rich plasma (PRP) as an augmentation in meniscus repair.

**Methods:**

A comprehensive search of PubMed, Medline (via EBSCO), ProQuest, and ScienceDirect from January to February 2023 was conducted using the terms “meniscus repair,” “PRP,” and “meniscus tear.” Meta-analyses that investigated the rate of failure after meniscus repair were included. Studies before 2003, not in English, associated procedures during surgery, and animal studies were excluded. The included studies underwent quality appraisal and risk of bias assessment. Data were extracted from each study‘s text, figures, tables, and associated supplementary files and then analyzed qualitatively.

**Results:**

The failure rate is lower in the PRP augmentation group compared with the group without augmentation, with a mean difference of 0.42, 0.50, and 0.43. Visual analog scale score was also found to be significantly lower in the treatment group, with a mean difference of 0.40, 0.76, and 6.69. However, only mean differences in Lysholm score in one of the included studies were found significant regarding functional outcomes, which can be found in the Xie et al. study with a mean difference of 3.06.

**Conclusions:**

In this study, we found that meniscal repairs augmented with PRP have a lower failure rate.

**Level of Evidence:**

Level IV, systematic review of Level III-IV studies.

Meniscal injury is one of the most common injuries in sports medicine, which is estimated at approximately 60 per 100,000 population, especially for those who practice physical contact sport.^1^, ^2^, ^3^, ^4^, ^5^ Meniscal tears are categorized by their shape and location. A tear within the outer one-third vascular zone, or “red-red” zone, has the potential for spontaneous healing, but when it comes to a tear in the two-thirds inner zone, “red-white” or “white-white” zone, a meniscal repair can be challenging because of the lack of blood supply.^2^^,^^3^ Based on its anatomy, the healing process of meniscus repair will be limited, with an incomplete healing process accelerating degeneration and leading to secondary osteoarthritis (OA).^2^, ^3^, ^4^, ^5^ Regenerative medicine such as mesenchymal stem cells or platelet-rich plasma (PRP) can be a breakthrough medicine for meniscal injury, especially in avascular meniscal injury.^2^^,^^6^^,^^7^

PRP is an autologous blood-derived product that contains 8 times more platelet held and multiple growth factors, such as transforming growth factor (TGF) β, platelet-derived growth factor, insulin-like growth factor, basic fibroblast growth factors, vascular endothelial growth factor, and many others. PRP promotes regenerative effects on cells, which can be seen in support of the tissue-healing process and repair cascade (inflammation, proliferation, and remodeling). Not only their regenerative effects, but PRP also has anti-inflammatory and antiapoptotic effects.^8^, ^9^, ^10^, ^11^

Previous meta-analyses tried to find the effectiveness of PRP as augmentation for meniscus repair. However, these results remain controversial.^12^, ^13^, ^14^, ^15^ The purpose of this study was to investigate the efficacy of PRP as an augmentation in meniscus repair. We hypothesized that the augmentation of PRP in meniscus repair will result in a lower rate of failure than that seen in patients who did not receive PRP.

## Methods

This study was performed and reported according to the Preferred Reporting Items for Systematic Reviews and Meta-analyses guideline.^16^ References to the included studies were also reviewed to fetch studies not found in the original search. The study protocol was registered in the PROSPERO International Prospective Register of Systematic Reviews. The PICO of this study was P (meniscal tear), I (meniscal repair augmented with platelet-rich plasma), C (meniscal repair), and O (rate of failure).

### Search Strategy

A thorough literature search was performed on PubMed, Medline (via EBSCO), ProQuest, and ScienceDirect from January to February 2023 with the following search string combination: “meniscus repair,” “platelet-rich plasma,” “meniscus tear.”

### Study Selection

All included studies contained original data published in English within 20 years. Meta-analyses that investigated the rate of failure after meniscus repair were included. Studies before 2003, not in English, associated procedures during surgery, and animal studies were excluded from the investigation.

### Quality Appraisal and Risk of Bias Assessment

Two independent authors (L.C.S. and S.A.K.) performed identification, selection, data extraction, and quality assessment. Different opinions between the 2 reviewers were resolved by reassessment and discussion with another author. The quality analysis of the literature was assessed with the Assessment of Multiple Systematic Reviews (AMSTAR). The AMSTAR consists of an 11-item checklist of reporting and methodology quality. The checklist includes instruments defining a meta-analysis’s weakness (critical or noncritical). The quality of the systematic review was defined as high (when there is no or 1 noncritical weakness), moderate (more than 1 noncritical weakness), low (1 critical weakness), and critically low (more than 1 critical weakness).^17^ The comprehensiveness of the systematic review and meta-analysis was also assessed using the QUOROM checklist, based on 17 items, with a point given when half of the criteria were met.^18^ The level of evidence was assessed using the Oxford Centre for Evidence-Based Medicine Guideline 2011.^19^

### Data Extraction and Analysis

Data were extracted from each study’s text, figures, tables, and associated supplementary files. These data included (1) article characteristics (journal, year of publication, PROSPERO registration, search string, search year, databases that included), (2) demographic characteristics (number of patients, number of patients with intervention and control), (3) quality assessment scale, and (4) outcomes (failure rate, revision rate, healing rate, International Knee Documentation Committee score, Lysholm score, and visual analog scale [VAS] score).

## Results

**Fig 1:**
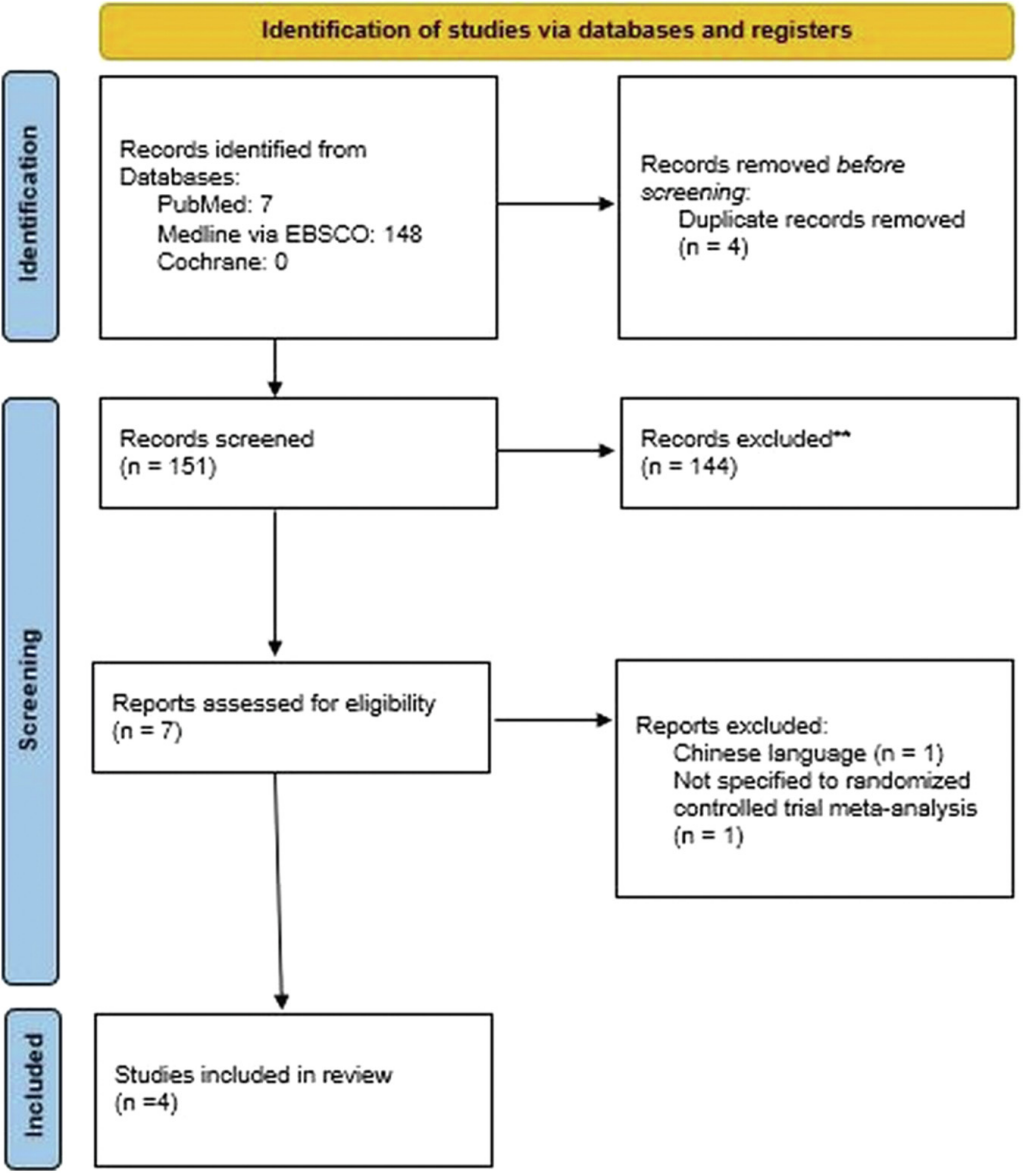
Preferred Reporting Items for Systematic Reviews and Meta-analyses flowchart.

A total of 155 studies were retrieved from the initial screening (Fig 1). In total, 151 studies were included after excluding duplication. Another 144 records were removed after the abstract screening. Of the remaining 7 studies, 1 did not use English,^20^ and the other did not do a meta-analysis from a randomized controlled trial, specifically Trams et al.^21^ study. The search strategy is summarized in Figure 1.

### Study Quality Assessment

The level of evidence was assessed using the Oxford Centre for Evidence-Based Medicine Guideline 2011, which found Levels III to IV of evidence in the included studies.^20^ The comprehensiveness of the systematic review and meta-analysis was evaluated using the QUOROM checklist described in Table 1. The score varies from 15 to 17 (with the maximum score being 17). The quality of the systematic review and meta-analysis was evaluated according to the AMSTAR quality assessment tool for systematic reviews and meta-analyses, where 3 of the included studies were considered high-quality studies, and 1 was regarded as a low-quality study, which is Xie et al.^12^ study (Table 2).

**Table 1:** QUOROM Checklist

No.	Heading	Subheading	Descriptor	Li et al.^13^	Migliorini et al.^15^	Wang et al.^14^	Xie et al.^12^
1		Title	Identify the report as a meta-analysis [or systematic review] of RCTs	v	v	v	v
2	Abstract	Objectives	The clinical question explicitly	v	v	v	v
3		Data sources	The databases (i.e., list) and other information sources	v	v	v	v
4		Review methods	The selection criteria (i.e., population, intervention, outcome, and study design); methods for validity assessment, data abstraction, and study characteristics and quantitative data synthesis in sufficient detail to permit replication	x	v	x	x
5		Results	Characteristics of the RCTs included and excluded, qualitative and quantitative findings (i.e., point estimates and confidence intervals), and subgroup analyses	v	v	v	v
6		Conclusion	The main results	v	v	v	v
7		Introduction	The explicit clinical problem, the biological rationale for the intervention, and the rationale for the review	v	v	v	v
8	Methods	Searching	The information sources, in detail (e.g., databases, registers, personal files, expert informants, agencies, hand-searching), and any restrictions (years considered, publication status, the language of publication)	v	v	v	v
9		Selection	The inclusion and exclusion criteria (defining the population, intervention, principal outcomes, and study design)	x	v	x	x
10		Validity assessment	The criteria and process used (e.g., masked conditions, quality assessment, and their findings)	v	v	v	v
11		Data abstraction	The process or processes used (e.g., completed independently, in duplicate)	v	v	v	v
12		Study characteristics	Study characteristics: the type of study design, participants’ characteristics, details of the intervention, outcome definitions, and how clinical heterogeneity was assessed	v	v	v	v
13		Quantitative data synthesis	The principal measures of effect (e.g., relative risk), method of combining results (statistical testing and confidence intervals), handling of missing data; how statistical heterogeneity was assessed; a rationale for any a priori sensitivity and subgroup analyses; and any assessment of publication bias)	v	v	v	v
14	Results	Trial flow	Provide a meta-analysis profile summarizing trial flow (see Fig 1)	v	v	v	v
15		Study characteristics	Study characteristics: present descriptive data for each trial (e.g., age, sample size, intervention, dose, duration, follow-up period)	v	v	v	v
16		Quantitative data synthesis	Quantitative data synthesis: report agreement on the selection and validity assessment; present simple summary results (for each treatment group in each trial, for each primary outcome); present data needed to calculate effect sizes and confidence intervals in intention-to-treat analyses (e.g., 22 tables of counts, means and standard deviations, proportions)	v	v	v	v
17		Discussion	Summarize key findings, discuss clinical inferences based on internal and external validity, interpret the results in light of the totality of available evidence, describe potential biases in the review process (e.g., publication bias), and suggest a future research agenda	v	v	v	v

RCT, randomized controlled trial; v, yes; x, no.

**Table 2:** Assessment of Multiple Systematic Reviews Quality Assessment

No	Instrument	Li et al.^13^	Migliorini et al.^15^	Wang et al.^14^	Xie et al.^12^
1	Did the research questions and inclusion criteria for the review include the components of PICO?	N	Y	N	N
2	Did the report of the review contain an explicit statement that the review methods were established prior to the conduct of the review and did the report justify any significant deviations from the protocol?	Y	Y	Y	Y
3	Did the review authors explain their selection of the study designs for inclusion in the review?	Y	Y	Y	Y
4	Did the review authors use a comprehensive literature search strategy?	Y	Y	Y	Y
5	Did the review authors perform study selection in duplicate?	Y	Y	Y	Y
6	Did the review authors perform data extraction in duplicate?	Y	Y	Y	Y
7	Did the review authors provide a list of excluded studies and justify the exclusions?	Y	Y	Y	N
8	Did the review authors describe the included studies in adequate detail?	Y	Y	Y	Y
9	Did the review authors use a satisfactory technique for assessing the risk of bias (RoB) in individual studies that were included in the review?	Y	Y	Y	Y
10	Did the review authors report on the sources of funding for the studies included in the review?	Y	Y	Y	N
11	If meta-analysis was performed, did the review authors use appropriate methods for statistical combination of results?	Y	Y	Y	Y
12	If meta-analysis was performed, did the review authors assess the potential impact of RoB in individual studies on the results of the meta-analysis or other evidence synthesis?	Y	Y	Y	Y
13	Did the review authors account for RoB in primary studies when interpreting/discussing the results of the review?	Y	Y	Y	Y
14	Did the review authors provide a satisfactory explanation for, and discussion of, any heterogeneity observed in the results of the review?	Y	Y	Y	Y
15	If they performed quantitative synthesis, did the review authors carry out an adequate investigation of publication bias (small study bias) and discuss its likely impact on the results of the review?	Y	Y	Y	Y
16	Did the review authors report any potential sources of conflict of interest, including any funding they received for conducting the review?	Y	Y	Y	N
	Quality	HIGH	HIGH	HIGH	LOW

N, no; Y, yes.

### Article and Demographic Characteristics

The characteristics of the 4 studies included in our study are shown in Table 3. A total of 2,614 patients were included in the current study. The studies were published from 2020 to 2022, with a sample size ranging from 293 to 837 patients.

**Table 3:** Characteristics of the Studies

No.	Authors	Journal	Year	PROSPERO	String	Search Year	Database						Included RCTs	Quality Assessment Scale
							PubMed	Web of Science	EMBASE	Medline	Cochrane Library	Other		
1	Li et al.^13^	*J Orthop Surg*	2022	Yes	((“PRP” OR “platelet-rich plasma” OR “plasma-rich fibrin”) AND (“meniscus” OR “menisci” OR “meniscal”))	Not stated	v	v	v	v	v		9	NOS
2	Migliorini et al.^15^	*J Orthop Traumatol*	2022	No	meniscal, menisci, augmentation, PRP, repair, combined, isolated, knee, arthroscopy, platelet-rich plasma, meniscopathy, damage, injury, tear, patient reported outcome measures, PROMs, Lysholm, IKDC, failure, complications, pain, revision, visual analog scale	Not stated	v	v	v			Google Scholar	8	Cochrane’s RoB
3	Wang et al.^14^	*J Int Med Res*	2020	No	platelet-rich plasma,” “thrombocyte-rich plasma,” “meniscus,” and “menisci”	Not stated	v		v		v		6	NOS
4	Xie et al.^12^	Medicina	2022	No	(platelet-rich plasma)AND(meniscus)	Not stated	v		v		v	ClinicalTrials.gov and CNKI	8	Cochrane’s RoB

IKDC, International Knee Documentation Committee; NOS, The Newcastle-Ottawa Scale; PROM, patient-reported outcome measures; PRP, platelet-rich plasma; RCT, randomized controlled trial; RoB, risk of bias; v, yes.

##### The Rate of Failure

Three studies evaluated the rate of failure after meniscus repair with and without PRP augmentation (Table 4). The failure rate is lower in the PRP augmentation group when compared to the group without augmentation according to 2 studies. Only 1 study found that there is no significant difference between the treatment and control groups.^12^, ^13^, ^14^, ^15^

**Table 4:** Failure Rate of Treatment and Control Group Among the Included Studies

No.	Authors	Year	Failure Rate				
			Treatment	Control	Results	95% CI	Heterogeneity, %
1	Li et al.^13^	2022	48/287	91/421	OR = 0.64, *P* = .03	0.42-0.96	0
2	Migliorini et al.^15^	2022	NA	NA	OR = 0.81, *P* = .42	0.50-1.34	0
3	Wang et al.^14^	2020	21/79	35/90	RR = 0.65, *P* = .03	0.43-0.97	31
4	Xie et al.^12^	2022	NA	NA	NA	NA	NA

NA, not applicable; OR, odds ratio; RR, risk ratio.

##### Pain Score

Among the 4 included studies, 3 studies conducted by Li et al.,^13^ Xie et al.,^12^ and Wang et al.^14^ found significant differences in VAS scores (Table 5). The result of the pain score shows that the VAS score in the PRP group is significantly lower than in the control group. However, high heterogeneity can be found.^12^, ^13^, ^14^, ^15^

**Table 5:** Visual Analog Scale of Treatment and Control Group Among the Included Studies

No.	Authors	Year	VAS				
			Treatment	Control	Results	95% CI	Heterogeneity, %
1	Li et al.^13^	2022	75	63	MD = –0.76, *P* = .007	–1.32 to –0.21	69
2	Migliorini et al.^15^	2022	NA	NA	MD = –0.05, *P* = .50	–0.18 to 0.09	84
3	Wang et al.^14^	2020	92	86	MD = –6.69, *P* < .001	–10.62 to –2.76	98
4	Xie et al.^12^	2022	126	124	MD = –0.40, *P* = .002	–0.66 to –0.15	9

CI, confidence interval; MD, mean deviation; NA, not applicable; VAS, visual analog scale.

##### Functional Score

Among the 4 included studies, only 1 study (Xie et al.^12^) found significant differences in the functional score, with the PRP group showing a higher enhancement in the functional score, which was assessed with the Lysholm score (Table 6). Three other included studies found no significant differences between the functional scores among the treatment and control groups.^12^, ^13^, ^14^, ^15^

**Table 6:** Functional Outcome (IKDC and Lysholm) of Treatment and Control Groups Among the Included Studies

No.	Authors	Year	IKDC					Lysholm				
			Treatment	Control	Results (MD)	95% CI	Heterogeneity, %	Treatment	Control	Results (MD)	95% CI	Heterogeneity, %
1	Li et al.^13^	2022	260	253	0.66, *P* = .16	–0.27 to 1.6	93	76	71	–0.13, *P* = .70	–0.78 to 0.52	69
2	Migliorini et al.^15^	2022	NA	NA	0.62, *P* = .91	–10.24 to 11.48	100	NA	NA	0.09, *P* = .99	–12.56 to 12.73	79
3	Wang et al.^14^	2020	89	76	1.96, *P* = .16	–0.73 to 4.72	97	50	67	0.08, *P* = .89	–0.99 to 1.15	87
4	Xie et al.^12^	2022	NA	NA	NA	NA	NA	156	166	3.06, *P* < .00001	1.70 to 4.42	15

CI, confidence interval; IKDC, International Knee Documentation Committee; MD, mean deviation; NA, not applicable.

## Discussion

This study shows that the PRP augmentation group had a lower failure rate and pain score than the control group. Regarding the functional outcome, there are no significant differences between the 2 groups.

The failure rate is the most common parameter that is used in studies. There are several definitions of failure rate, which can be seen through second-look arthroscopy evaluation. Kaminski et al.^22^ defined treatment failure as no visible healing during a second-look arthroscopy or less than 50% healing of the tear width versus unstable repair on magnetic resonance imaging review. Meanwhile, healing rate is defined by the number of fully healing menisci plus partially healing menisci divided by the total meniscus assessed in the intervention group.^23^ When entire meniscus integrity was observed during magnetic resonance arthrography (no intrameniscal contrast agent), complete healing was taken into consideration. Compartment media were used to fill up a 1- to 3-mm defect to evaluate partial healing.^23^, ^24^ This systematic review shows that PRP is used to reduce the risk of failure or collapse. A study by Xie et al.^12^ also showed that PRP use has a superior effect on the healing rate compared with the control group. This positive effect of healing rate was observed 24 to 33 weeks after injection of PRP. Meniscus is composed of 72% of water and 28% of organic structures such as collagen (75%), and it is primarily composed of type I collagen, glycosaminoglycans (17%), DNA (2%), adhesion glycoproteins (<1%), and elastin (<1%).^1^^,^^17^^,^^18^ Growth factors are the most prominent biochemical stimuli for meniscal engineering, while PRP augmentation can help to stimulate the growth hormones.^21^, ^25^, ^26^, ^27^ The understanding theory of PRP augmentation in meniscal injury is the growth factors that PRP release promoter proliferation of meniscus cells on any zone of meniscal and increasing collagen synthesis. TGF-β1 also provides expression and secretion of lubricin or superficial zone protein, which provides lubrication of cartilage.^25^

PRP use in meniscal injury has positive results in pain score and a lower rate of failure than in control groups. Pain score, evaluated by VAS score, showed that the PRP group had improved in controlling the pain better than control groups. This is a result of PRP containing growth hormones promoting anti-inflammatory effects. Inflammation factors (interleukin [IL] 1β, IL-6, and tumor necrosis factor [TNF] α) are released after stress and surgery, which decrease the threshold of the nociceptor, and so there is pain. Many growth factors contained in PRP can inhibit the local inflammatory response. PRP also influences the expression of prostaglandin E2, substance P, dopamine, and 5-hydroxy-tryptamine to reduce pain. In meniscal injury, fibroblast growth, platelet-derived growth hormones, and TGF-β1 may contribute to scar formation, especially in the red-red zone, whereas the avascular zone, white-white zone, and PDGF-AB contribute well to healing. Platelets in PRP will release multiple anti-inflammatory cytokines, including IL-1 receptor antagonist, soluble TNF receptors I and II, IL-4, IL-10, IL-13, and interferon γ. These anti-inflammatory cytokines inhibit bioactivity of inflammatory cytokine, preventing its signal transduction, and reduced prostaglandin E2 production. PRP also has a role in 5-HT, which has a critical role in pain tolerance. However, these findings are not consistent, because in the study conducted by Xie et al.,^12^ the PRP group only showed a better effect in month 6, not in months 1 and 12.

The International Knee Documentation Committee and Lysholm scores are used to evaluate the postoperative recovery of knee function. Irrgang et al.,^26^ in 2006, suggested that a change in score of 11.5 points had the highest sensitivity, and a change in score of 20.5 points had the highest specificity to define a successful improvement in knee function.^28^ In this systematic review, the knee function had no significant difference between the PRP group and control groups. An in vivo investigation using PRP and bone marrow-derived mesenchymal stem cells (BMSCs) to enhance white-white zone injury in beagle dogs showed that healing on knee meniscus in the control group was 0 of 20, BMSC group 1 of 20, PRP group 9 of 20, and PRP + BMSC group 10 of 20.^27^ This result indicated that PRP increases the expression of type I and II collagen^27^, ^29^ during the meniscus injury repair process and reduces osteopontin concentration.^27^, ^29^ Osteopontin is a kind of phosphoprotein that promotes bone turnover and remodeling and accelerates the progression of OA.^30^ Wang et al.^14^ also showed that the PRP group’s range of active knee flexion has significantly improved more than in the control groups. This may be because PRP releases many growth factors and anti-inflammatory cytokines to promote cell proliferation and regulate cell behavior to reduce local inflammation, thus reducing swelling and effusion of the knee and reducing pain.^8^, ^9^, ^10^, ^11^

In a meta-analysis, Xie et al.^12^ reported 1 study that had adverse effects of PRP use. In that study, 2 people had mild joint swelling, pain, and limited mobility, which disappeared 3 days after ice compress, movement restriction, and administration of oral analgesics.

The strength of the study was to evaluate the results of other pre-existing meta-analyses. Various database searches from 4 databases also increased our study searching quality.

### Limitations

This study is not without limitations. The main shortcomings of this study are a direct result of the shortcomings of the available data. Only 4 meta-analyses met inclusion criteria, and these were highly heterogeneous. This reduces the validity of our results.

## Conclusions

In this study, we found that meniscal repairs augmented with PRP have a lower failure rate.

## Disclosures

The authors declare that they have no known competing financial interests or personal relationships that could have appeared to influence the work reported in this paper.

## Supplementary Data

ICMJE author disclosure forms
