# Supplementary material for: The Use of Platelet-Rich Plasma Augmentation in Meniscus Repair Results in a Lower Failure Rate than in the Control Group: A Systematic Review From Meta-analysis
Source: Arthrosc Sports Med Rehabil. 2024 Apr 9;6(4):100934. doi: 10.1016/j.asmr.2024.100934 (PMC11480798; doi:10.1016/j.asmr.2024.100934)
Supplement: ICMJE author disclosure forms [file mmc1.docx]

**Muhammad Sakti, MD, Ph.D.**

**Declaration of interests**
 
☒ The authors declare that they have no known competing financial interests or personal relationships that could have appeared to influence the work reported in this paper.
 
☐ The authors declare the following financial interests/personal relationships which may be considered as potential competing interests:

**Prof. Andi Idrus Paturussi, MD, Ph.D.**

**Declaration of interests**
 
☒ The authors declare that they have no known competing financial interests or personal relationships that could have appeared to influence the work reported in this paper.
 
☐ The authors declare the following financial interests/personal relationships which may be considered as potential competing interests:

**Leonard Christianto Singjie, MD**

**Declaration of interests**
 
☒ The authors declare that they have no known competing financial interests or personal relationships that could have appeared to influence the work reported in this paper.
 
☐ The authors declare the following financial interests/personal relationships which may be considered as potential competing interests:

**Samuel Andi Kusuma, MD**

**Declaration of interests**
 
☒ The authors declare that they have no known competing financial interests or personal relationships that could have appeared to influence the work reported in this paper.
 
☐ The authors declare the following financial interests/personal relationships which may be considered as potential competing interests:

 
 
 
